# Health outcomes and the transition experience of HIV-infected adolescents after transfer to adult care in Québec, Canada

**DOI:** 10.1186/s12887-016-0644-4

**Published:** 2016-07-26

**Authors:** Fatima Kakkar, Dimitri Van der Linden, Silvie Valois, Francois Maurice, Marion Onnorouille, Normand Lapointe, Hugo Soudeyns, Valerie Lamarre

**Affiliations:** 1 Division of Infectious Diseases, CHU Sainte-Justine, 3175 Côte Sainte-Catherine, Montréal, Québec H3T 1C5 Canada; 2 Department of Pediatrics, Faculty of Medicine, Université de Montréal, Montréal, Canada; 3 Cliniques universitaires Saint-Luc, Maladies Infectieuses Pédiatriques, Service de Pédiatrie Générale, Bruxelles, Belgium; 4 Centre Maternel et Infantile sur le SIDA, CHU Sainte-Justine, Montréal, Canada; 5 Unité d’immunopathologie virale, Centre de recherche du CHU Sainte-Justine, Montréal, Canada; 6 Department of Microbiology, Infectious Diseases and Immunology, Faculty of Medicine, Université de Montréal, Montréal, Canada

**Keywords:** HIV, Perinatal infection, Transition, Adolescents, Outcomes

## Abstract

**Background:**

Little is known on outcomes after transition to adult care among adolescents with perinatal HIV infection. Though there is data from other chronic pediatric diseases suggesting increased morbidity and mortality following transfer to adult care, this has not well been studied among the first wave of survivors of perinatal HIV infection. The primary objective of this study was to determine outcomes after transition to adult care among a cohort of HIV-infected adolescents in Québec, Canada. Secondary objectives were to document participant experiences with the transition process, identify barriers to successful transition, and potential changes to improve the transition process.

**Methods:**

Clinic records were reviewed to identify all perinatally-infected youth who transitioned from the Centre Maternel et Infantile sur le Sida pediatric HIV clinic (Montreal) at age 18 to an adult care provider between 1999 and 2012. Transitioned patients were contacted using last available patient or parental listed phone number on hospital record, internet based telephone directory, or social media. A standardized questionnaire was administered by telephone or in-person interview, and copies of current medical records obtained from treating physicians.

**Results:**

Forty-five patients were transferred between 1999 and 2012, among whom 25 consented to the study, eight were lost to follow-up, eight refused participation, and four were deceased. Overall 76 % of patients remained engaged in care, defined by at least one physician visit within 6 months of the interview. Over 50 % reported difficulty with adherence to their current drug regimens. At one-year post-transfer, there was a decrease in the proportion of patients with CD4 count >500 cells/mm^3^ from 64 to 29 %, and a statistically significant decrease in absolute CD4 count (mean 370 vs 524 cells/mm^3^, *p* = 0.04.). The majority (92 %) of participants felt that 18 was too young an age to transfer to adult care, and provided suggestions for improving the transition process.

**Conclusions:**

This group of perinatally-infected youth remained engaged in care after transition, however difficulties with adherence and assuming responsibility for their own care were identified as issues in their post-transition care. The high rate of mortality among them and the changes to their health status post-transition suggest that further work is necessary to document the health outcomes of this group in larger, more diverse cohort settings.

## Background

The success of combination antiretroviral therapy (cART) has changed the course of pediatric HIV from a once fatal disease to a chronic one [1]. Worldwide, increasing numbers of perinatally-infected children have reached the age at which they will need to transition from pediatric to adult care. Transition, formally defined as the “purposeful, planned movement of adolescents with chronic medical conditions from child-centered to adult-oriented health care” [2], has historically been challenging [3, 4]. Though there is a wealth of information from other chronic pediatric diseases that illustrates that the transition process is often associated with increased morbidity, mortality, poorer social and educational outcomes, and high rates of loss to follow-up and non-retention in care [5–7], adolescents with HIV have unique characteristics when compared to other pediatric populations, which make it difficult to extrapolate data from transition studies of other chronic diseases [8].

While the normal transition process is marked by a sense of loss and grief that occurs when going from one life stage to another, this change may be experienced more intensely for young adults living with HIV, as many have already suffered the loss of important caregivers in their lives, including the death of one or more parent [9]. The break of ties with pediatric staff with whom they may have developed longstanding relationships may represent additional loss [10, 11]. Moreover, as a chronic illness, HIV is unique because of HIV-related stigma, the relationship to poverty, and the fact that multiple members of the same family may be living with or have died from HIV infection [12, 13]. Emerging data on outcomes after transition among perinatally-infected youth is concerning; a recent study from the US HIV Research Network found that 19.8 % of transitioned 21 year-olds were lost-to-follow-up after their 22^nd^birthday [14], and had lower prevalence of virological suppression compared with horizontally-infected young adults [15]. A similar study among 20–24 year olds in the UK found a significantly higher mortality rate compared to HIV uninfected youth [16].

The Centre Maternel et Infantile sur le Sida (CMIS) cohort was established in 1988 to follow all HIV-infected children in care at Centre Hospitalier Universitaire (CHU) Sainte-Justine, a tertiary care hospital, in Montréal, Canada. Under a system of universal health care access, children received cART as soon as it became available in 1997 and were followed until age 18, at which point they were transferred to adult care. We previously described the clinical and immunological status of these adolescents at the time of transfer [17], and found alarmingly that over two thirds were failing treatment, manifested by detectable viremia, CD4 counts <200 cells/mm^3^, and/or triple class resistance. Given concerns about their health status prior to transition, and the paucity of data on post-transition outcomes, the primary objective of this study was to determine outcomes after transition to adult care among this cohort of HIV-infected adolescents in Québec, Canada, a province with one of the youngest ages of transition in the developed world. Secondary objectives were to document participant experiences with the transition process, identify barriers to successful transition, and potential changes to improve the transition process.

## Methods

### Participants

Clinic records were reviewed to identify all youth who were transferred from the CMIS to an adult care provider between 1999 and 2012. Inclusion criteria for the study included 1) Perinatal HIV infection 2) Engaged in care prior to transfer (attendance at least 3 appointments per year in the 2 years prior to transfer) 3) Capacity to communicate (verbally or written), and 4) Elapsed time of at least 1 year since transfer. After the study was approved by the Institutional Review Board of CHU Sainte-Justine Research Center, patients were contacted by the CMIS research nurse for consent to enter into the study using last available patient or parental listed phone number on hospital record, internet based telephone directory (Canada 411), or social media (Facebook).

### Procedure

For those who consented to the study, a semi-structured standardized questionnaire was administered by telephone or in-person interview by the CMIS research nurse, and permission obtained to contact their current treating physicians for their medical records. Participants interviewed by telephone were offered a gift card of 50$ to compensate them for their time, and those interviewed in person were supplemented with $20 to compensate for travel. In addition to the structured questionnaire, participants were asked the following three questions to describe their experience with the transfer of care.

1. Do you feel that you were ready to transfer to adult care at age 18?
2. Are you satisfied with your new adult care provider? Why or why not?
3. Do you have suggestions for improving the transition process?

Responses were transcribed verbatim and entered into a central database after which they were translated into English. Responses were grouped by theme according to the questions asked, and quoted in the text to illustrate the diversity of answers (those which were repeated were not quoted). Descriptive statistics were used to describe the study population, and paired-t-test was used to compare pre-and post-transition CD4 count. All data was analyzed using STATA vs.13.0.

### The transition process

By age 18, the care of HIV-infected children was officially transferred to an adult care provider. Patients were given a choice of an adult care provider closest to them, and were accompanied to their first visit by the pediatric clinic social worker. At the time of the study there were no other interventions in place to facilitate transfer of care. A complete medical and psychosocial summary of the patient was sent to the new adult treating physician. After transfer, no further medical care was provided at CMIS unless pregnancy occurred, at which point the patients were re-engaged by the obstetrics team at CHU Sainte-Justine and their newborn infants followed at CMIS for prevention of mother-to-child transmission.

## Results

Fifty-four patients were transferred from the pediatric clinic to an adult care provider between 1999 and 2012, among whom 45 were eligible for study. Reasons for exclusion included horizontal transmission (1), disengaged in care prior to transfer (2), unable to communicate (verbally or written) (3), and less than 1 year elapsed since transfer (3). Among 45 eligible patients, 25 successfully completed the study; 8 refused participation, four were deceased at time of study, and eight could not be reached. Among the eight who were not successfully contacted, four were confirmed alive by family members or their current physicians. All of the patients confirmed deceased were transferred between 1999 and 2002.

Characteristics of the participants are described in Table 1. Mean time that had elapsed between transfer and interview was 3.6 years (range 1.1–6.8 years), and mean age at time of interview was 22 years (range 19–25 years). Sixty percent of the interviews were conducted in person, 40 % by telephone. At the time of transfer, 92 % were still living with at least a parent and other family member. Most (88 %) of the youth were accompanied to their first adult appointment by the pediatric clinic social worker or a family member. At the time of interview, the same percentage reported that they were now going to their appointments by themselves. Seventy-six percent remained engaged in care, defined by at least one physician visit within 6 months of the interview (as per standard Québec adult HIV treatment follow-up guidelines) [18]. Fifty-two percent had obtained a high school degree or higher, 64 % were still in a school setting, and 33 % had completed their training to date. Among those still in school, 50 % were also working part-time. Twenty-five percent of the youth were living on social assistance (Canadian welfare program).

**Table 1:** Post-Transition Patient Characteristics

Variable	*n* (%)
Gender	
Male	10 (40)
Female	15 (60)
Ethnicity	
Canadian Born	17 (68)
Foreign Born	8 (32)
Presently in School Setting	
Yes	16 (64)
No	9 (36)
Highest degree Obtained	
No High School	7 (28)
High School	9 (36)
Professional degree	5 (20)
Pre college	4 (16)
University	0
Extended family without parents	7 (28)
Alone or with a partner	2 (8)
Living with at time of transfer	
At least one parent	16 (64)
Last physician follow-up	
Within past 6 months	19 (76)
Within past 12 months	3 (12)
Not for over 1 year	3 (12)

### Medical outcomes

Prior to transfer, 64 % of patients had an absolute CD4 count greater than 500 cells/mm^3^, 16 % between 200 and 500 cells/mm^3^, and 20 % were immunosuppressed with a CD4 count <200 cells/mm^3^. This changed at 1 year post-transfer, with a decrease in the proportion of patients with CD4 counts >500 cells/mm^3^ to only 29 %, and a corresponding increase in the proportion with CD4 counts between 200 and 500 cells/mm^3^ and <200 cells/mm^3^ (41 % and 29 %, respectively). This represented a statistically significant decrease in absolute CD4 count (mean 370 vs. 524 cells/mm^3^, *p* = 0.04). With respect to VL, prior to transfer, 60 % of patients had an undetectable viral load VL (<40 copies/ml), while among those who were detectable, all had VL>1000 copies/ml. Among 16 patients for whom VL measures were available at 1 year, of the ten who had had undetectable VL prior to transfer, nine remained undetectable, and one increased VL (<40 to 1759 copies/ml). All six patients with detectable VL prior to transfer remained detectable at 1 year post-transfer. At the time of the interview, only seven youth remained on the same regimen they had been on at the time of transfer, 11 had changed regimen, two had stopped all ARV therapy, three who had not been on treatment initiated therapy, and two still were not on treatment. When asked how often they had missed drug doses in the previous month, only 40 % of patients reported excellent adherence to their current drug regimens (no missed doses), 28 % reported occasional missed doses, 16 % reported frequently missed doses, and 12 % had stopped all ARV therapy.

### Pregnancy, sexual health, and disclosure (Table 2)

**Table 2:** Sexual Practices, Disclosure and Pregnancy among Transitioned Youth

Response to questionnaire	*N* (%)
I have regular sexual partners	
Yes	20 (80)
No	5 (20)
I routinely disclose my HIV status to my regular sexual partners	
Yes	13 (65)
No	7 (35)
I always use condoms with my regular sexual partners	
Yes	12 (60)
No	8 (40)
I have occasional sexual partners	
Yes	12 (48)
No	12 (52)
I disclose to my occasional sexual partners	
Never	10 (83)
Occasionally	2 (17)
I always use condoms with my occasional sexual partners	
Yes	10 (83)
No	2 (17)
I have been diagnosed with a sexually transmitted infection since the time of transfer	
Yes	5 (25)
No	20 (75)
Has become a parent (all respondents)	
Yes	5 (25)
No	20 (75)
Ever pregnant (among females *n* = 15)	
Yes	6 (40)
Termination	3 (50)
Live birth	3 (50)

Seventy-two percent of youth were self-described as single, not in any significant relationship (defined as dating, married or living with their partner). Overall, 25 % had become parents themselves (three women, two men). Eighty percent of the youth stated that they had regular sexual partners, to whom only 65 % routinely disclosed their HIV status. Among those with occasional sexual partners (*n* = 12), the majority (83 %) did not disclosure their HIV status to them. While only 60 % answered that they always used condoms with their regular sexual partners, 83 % stated they always used condoms with their occasional sexual partners.

### Reflections on the transition process

When asked whether they had felt ready to transition to adult care at age 18, the majority of participants (92 %) stated that they weren’t ready to transition at the age of 18, and would have preferred the option of staying longer under pediatric care. Specific comments on the age of transition are listed below:

*“I wasn’t ready, I would have preferred staying until at least I knew how to make my own appointments and go by myself. Here everyone helped, afterwards I was on my own”. (19 year-old)*

*“18 was way too early. I wasn’t ready and I had a lot of trouble, I had to go a whole month without my medications because there was no one to help get them, and my viral load went up”. (23-year-old)*

*“It was too much of a change too fast. 18 is too young to leave kids all by themselves. Suddenly we were given all new responsibilities, I had to my make my own appointments and it was hard. I was used to the old system where we had lots of help”. (19 year-old)*

### Suggestions on improving the transition process

Participants were then asked for suggestions on how to improve the transition process. Themes that emerged included allowing youth to maintain ties to non-medical members of pediatric team and other youth in clinic, alternating appointments between the adult and pediatrics doctors until solid ties to the adult doctor had been established, and being provided with more information on what was going to happen in the adult clinics. Specific comments are below:

*“ I would have liked to have visited the adult doctor a few times, while still having medical appointments at CMIS, until I was sure I felt comfortable with him and I knew how to make my appointments and where to go”. (24 year-old)*

*“ I would have preferred to have something in-between, maybe an 18–25 year old clinic”. (19 year-old)*

*“I would have preferred to try different adult doctors until I found one that was right for me”. (24 year-old)*

*“I was ok with the transfer of my medical appointments to someone else, but I really would have preferred to be able to keep my social worker until I could find someone else to help”. (24 year-old)*

*“We should have been given more responsibility when we were younger – around 16 – to do the things we need to do on the adult side like making our own appointments”. (20 year-old)*

### Reflections on their experience with adult care providers

Participants were then asked to describe their experience with their new adult care providers and any difficulties they were having. Their comments reflect a sense of dissatisfaction with the rapidity of appointments, the feeling of being rushed and unable to connect to adult care providers.

*“The appointments go too fast on the adult side and they give you little information. I would have liked more information before I left, about how the appointments would be, what kind of treatments there would be”. (22 year-old)*

*“It (the appointment) goes too quickly on the adult side – you’re done in 20 minutes, blood taken, the doctors asks you how you are doing and gives you your prescription. There isn’t any time to talk. They don’t explain things”. (20 year-old)*

*“I feel the adult doctor doesn’t really care if I show up or not” (21 year-old)*

*“The problem is that the visit only lasts for 5 min and you only get to see the doctor. No one else seems to care”. (23 year-old)*

## Discussion

This study is the first to report on post-transition outcomes among perinatally-infected youth in Montréal, Canada, in a province with one of the youngest ages of transition in the developed world. Though only 25 patients participated (just over 50 % of those eligible), the results highlight some common issues and some major concerns in their post-transition care.

First, with respect to their overall medical status, it is concerning that nearly a quarter of transitioned patients were no longer engaged in care. This was reflected in the change in their health status post-transfer, with a notable decrease in their CD4 counts. In addition to lack of engagement in care, we suspect that the changes seen to CD4 count may also reflect difficulties with adherence and drug procurement that have occurred post-transition, which has previously been described among transitioned youth in the United States [19]. While all young adults are covered by the provincial health care plan in the province of Québec, participation in the system requires ongoing registration, which many found difficult to do on their own. Moreover, the same medications which were free of charge while they were “children” (under age 18) now require a monthly co-payment. Adult clinics receiving these young adults have expectations that they are able to assume responsibility for their own appointments, drug coverage and costs, tasks that many adolescents are not equipped for at the time of transfer. We were further surprised at the low rate of excellent adherence (by self-report) among the transitioned teens. The use of self-report to measure adherence is generally biased upwards due to both recall bias and the desire of respondents to please interviewers [20]. In this respect, a self reported excellent adherence rate of 40 % is concerning. Moreover, the participants in the study likely represent those most engaged in care and thus likely to be adherent, perhaps overestimating the true rates of excellent adherence.

Perhaps most concerning is that the overall mortality among this cohort of transitioned teenagers was, at the very least, 8.8 %. Though this is higher than that mortality rate recently reported among a cohort of transitioned teens from the United Kingdom (0.9 per 100 person-years) [16], it is consistent with global reports citing an increase in the number HIV-related deaths among adolescents, with HIV/AIDS now the second leading cause among adolescents worldwide [21]. While difficulties with the transition process may contribute to this, we suspect that this likely reflects their poor health status prior to transition while still under pediatric care. Given their high rates of drug resistance and poor immunological status prior to transition [17], it is not surprising that these same young adults may be failing therapy under adult care. We suspect that those with difficulties prior to transition continue have difficulties post-transition, and that the challenges brought on by the transition process may worsen the already fragile health status of some. As much as efforts should be made to improve the transition process, the health and social welfare of these teenagers must be optimized prior to transition.

With respect to the transition experience, an overall theme that emerged was the difference in adult and pediatric care practice styles, and the resulting difficulty youth had in adapting. Pediatric care was more flexible with respect to appointments, with frequent reminders for missed visits, help with transportation to and from clinic, and with obtaining medications. The loss of these services was challenging for those who did not have the tools to adapt. These results concur with previous qualitative studies identifying barriers in the transition process [22–25], including lack of communication between pediatric and adult care providers [26], lack of developmental readiness [27], and fear of attending clinics with adults with horizontally-acquired HIV [23]. The youth respondents in this study provided insight into ways of improving the transition process, with suggestions that included increasing their preparedness for the process with increased responsibility prior to transfer, a period of shared care between adult and pediatric providers until successful transition had occurred, a special young adult (18–25) clinic, and the possibility of maintaining links to non-medical members of the pediatric team. A delayed transition was also proposed by some, with a preference towards 18–20 or until judged by both patient and physician to be “ready”.

## Conclusions

This study among a small group of perinatally-infected youth after transfer to adult care demonstrates that while a majority remained engaged in care after transition, difficulties with adherence and assuming responsibility for their own care were identified as major issues in their post-transition care. Our results must be interpreted with caution, as they may not be reflective of the overall transitioned cohort of young adults. Those participants we were unable to recruit likely represent those at highest risk of adverse outcomes, and represent the major limitation of this study. Moreover, our small sample size makes it difficult to generalize our findings. The outcomes of those whom we were unable to contact or who refused to participate may well be very different than those reported, and yet are an important group who may most benefit from any interventions. Finally, the youth participants in this study were those who grew up in an era of limited cART availability, and their health outcomes may be very different from those perinatally-infected youth currently engaged in care. Nonetheless, given the limited data available on post-transition outcomes, our study does provide some insight into the post-transition world of HIV-infected youth, and suggests that further efforts should be directed towards systematically following perinatally-infected youth and their trajectory into adulthood, in order to best guide current pediatric care and the transition process.

## Abbreviations

cART, combination antiretroviral therapy; CHU, Centre hospitalier universitaire; CMIS, Centre maternel et infantile sur le SIDA; HIV, human immunodeficiency virus type 1
